# Differential Proteomic Analysis of Mammalian Tissues Using SILAM

**DOI:** 10.1371/journal.pone.0016039

**Published:** 2011-01-20

**Authors:** Daniel B. McClatchy, Lujian Liao, Sung Kyu Park, Tao Xu, Bingwen Lu, John R. Yates III

**Affiliations:** Department of Chemical Physiology, The Scripps Research Institute, La Jolla, California, United States of America; University of Cambridge, United Kingdom

## Abstract

Differential expression of proteins between tissues underlies organ-specific functions. Under certain pathological conditions, this may also lead to tissue vulnerability. Furthermore, post-translational modifications exist between different cell types and pathological conditions. We employed SILAM (Stable Isotope Labeling in Mammals) combined with mass spectrometry to quantify the proteome between mammalian tissues. Using ^15^N labeled rat tissue, we quantified 3742 phosphorylated peptides in nuclear extracts from liver and brain tissue. Analysis of the phosphorylation sites revealed tissue specific kinase motifs. Although these tissues are quite different in their composition and function, more than 500 protein identifications were common to both tissues. Specifically, we identified an up-regulation in the brain of the phosphoprotein, ZFHX1B, in which a genetic deletion causes the neurological disorder Mowat–Wilson syndrome. Finally, pathway analysis revealed distinct nuclear pathways enriched in each tissue. Our findings provide a valuable resource as a starting point for further understanding of tissue specific gene regulation and demonstrate SILAM as a useful strategy for the differential proteomic analysis of mammalian tissues.

## Introduction

A puzzling phenomenon in many neurological diseases is that mutations in individual genes cause neurological specific phenotypes, but the genes are ubiquitously expressed throughout the body. It has been proposed that post-translational modifications specific to one tissue may generate tissue specific functions for a given protein. This has been demonstrated for methyl-CpG-binding protein 2 (MECP2). MECP2 is a transcriptional repressor through binding to methylated DNA, and mutations in this protein cause the majority of the cases of Rett syndrome(RTT) [1], [2], [3]. RTT is an X-linked neurodevelopmental disorder and is a leading cause of mental retardation in females [4]. Although MECP2 is ubiquitously expressed, it has been demonstrated that it is phosphorylated at S421 only in the brain, and this neuronal specific phosphorylation event leads to the transcription of brain-derived neurotrophic factor (BDNF) [5], which is crucial for neuronal cell development and neural circuits formation. Although this MECP2 study is a breakthrough in the role of phosphorylation in neurological disease, it is tempting to speculate that other phosphorylation events might happen in MECP2 as well as other master regulatory proteins during cell differentiation and tissue development that contribute to pleiotropic functions. However, there has been no quantitative large-scale analysis of the phosphorylation differences between the brain and other mammalian tissues.

Protein phosphorylation has been studied extensively on an individual basis, but there is an emerging trend to study phosphorylation on a proteomic scale. Global analysis of protein phosphorylation using tandem mass spectrometry (MS/MS) is beneficial in several aspects. First, since MS/MS combined with database searching algorithms directly derives sequence information of peptides, it is therefore capable of identifying novel phosphorylation sites [6], [7], [8]. Second, bioinformatic analysis of a large number of phosphopeptides can help extract consensus sequences indicating the kinase responsible for the phosphorylation [9], [10], [11]. Finally, mass spectrometry data is quantitative so differences in the relative expression of phosphorylation events between samples can be calculated.

Quantification can be achieved by comparing a peptide with an identical peptide that is labeled with heavy isotopes (e.g. ^13^C or ^15^N) [12], [13]. Given that a mass spectrometer can recognize the mass difference between light and heavy peptides, an abundance ratio between the labeled and unlabeled peptides can then be calculated from the respective ion chromatograms [14], [15]. To label a protein sample with stable isotopes, either metabolic or in vitro labeling can be employed [16], [17]. Alterations in protein expression induced by a stimulus can be determined by analyzing two samples utilizing the same labeled internal standard [14]. Metabolic labeling has advantages over in vitro labeling techniques since it exploits the cell's translational machinery to label all the proteins, while some in vitro labeling techniques use chemical reactions to label proteins with only certain functional groups[18]. In addition, in vitro labeling techniques label peptides after digestion, and then the light and heavy samples are mixed, while metabolic labeling allows for the mixture of light and heavy samples before any sample preparation, such as the isolation of a specific organelle. Thus, metabolic labeling reduces the systematic errors that may accumulate during sample preparation between the heavy and light samples [14]. Metabolic labeling is routinely used in simple systems, such as yeast and cultured mammalian cells and has even been applied to simple organisms, including *C. elegans* and *D. melanogaster*, to quantify hundreds to thousands of unmodified and phosphorylated peptides [19], [20]. In comparison, few studies have performed large-scale quantitative phosphorylation analysis on mammalian tissue, and those that have employed *in vitro* labeling techniques [21], [22]. In order to study animal models of disease, we developed the strategy SILAM (Stable Isotope Labeling of Mammals) to metabolically label an entire mammal for quantitative MS analysis [23], [24]. This strategy combines the necessity of studying mammalian tissues with the quantitative advantage of metabolic labeling. We previously demonstrated that labeling a rat with ^15^N for two generations had no adverse health effects and generated an entire animal highly enriched with ^15^N that was phenotypically normal [24]. We validated the SILAM strategy by quantifying alterations in unmodified peptides in liver tissue induced by a systemic injection of cyclohexamide and in brain tissue during postnatal development [23], [25].

We propose that SILAM can be employed to quantitatively compare the proteomes of different tissues. To validate our strategy, we quantified differences between the liver and the brain proteomes. The liver plays a major role in metabolism and has a number of other functions in the body, including glycogen storage, decomposition of red blood cells, and detoxification. The major cells that carry out these functions are hepatocytes. In addition, the liver is capable of regeneration. In contrast, the brain is incapable of regeneration and controls movement, perception, and cognition to generate complex behaviors. The brain consists of terminally differentiated neurons and smaller dividing glia. We chose to examine the nuclear proteome of these tissues, because although the fundamental functions of the nucleus are similar in all cells, nuclear proteins produce a variety of specific cellular characteristics through differential control of gene expression.

## Materials and Methods

### Nuclear enriched sample preparation

Sprague-Dawley rats were labeled with ^15^N as previously described [23], [24]. Briefly, a female rat was fed a ^15^N labeled protein diet starting after weaning, remaining on the ^15^N protein diet throughout its pregnancy, and while feeding its pups. On postnatal day 45 (p45), the pups were subjected to halothane by inhalation until unresponsive, and the tissues were quickly removed, frozen with liquid nitrogen, and stored at –80°C. The ^15^N enrichment was determined to be 96% using a previously described protocol[26]. Livers and brains from unlabeled Sprague-Dawley rats at p45 were obtained and stored in an identical manner as the ^15^N labeled brains. All methods involving animals were approved by the Institutional Animal Research Committee (approval #07-0083) and accredited by the American Association for Accreditation of Laboratory Animal Care.

Three snap-frozen p45 rat livers and brains, as well as ^15^N enriched rat liver were homogenized in a buffer (1 g of tissue/10 ml of buffer) containing 4 mM HEPES, 0.32 M sucrose, protease and phosphatase inhibitors(Roche, Indianapolis, IN) in a Teflon hand held dounce grinder. Before homogenization, the rat livers were minced with a razor blade and then further grounded with an Omni Tissue Master 125 electric grinder. After determining the protein concentration with a BCA protein assay (Pierce, Rockford, IL), homogenates from either liver or brain were mixed at a 1∶1(wt/wt) ratio with the ^15^N liver homogenate. The nuclei were isolated following a previous published protocol [27]. Briefly, the ^14^N/^15^N mixture was added to 10 ml of buffer and then was centrifuged at 800× g for 15 minutes. The resulting pellets were resuspended in 1 ml of buffer containing 0.5% NP-40, and then, incubated on ice for 2 hours. The lysate was added to 10 ml of buffer and centrifuged at 800× g for 15 minutes. The resulting pellets were homogenized in 500 ul of buffer and protein concentration was determined with a BCA protein assay. In total, this resulted in three ^14^N liver/^15^N liver nuclear preparations and three ^14^N brain/^15^N liver. Verification of the purity of this nuclear preparation by western blot analysis has been previously published [28].

### Trypsin digestion and enrichment of phosphopeptides using Immobilized Metal Affinity Chromatography (IMAC)

One milligram of each ^14^N/^15^N mixture was precipitated with trichloroacetic acid at a final concentration of 20% for 30 minutes and washed twice with cold acetone. The pellets were then solublized by sonication with 100 ul 5x Invitrosol (Inivtrogen, Carlsbad, CA) with 4M urea, reduced with 10 mM dithiothreitol and alkylated with 10 mM iodoacetamide for 30 minutes at room temperature, respectively. The solutions were diluted with 4x volumes of 100 mM Tris-HCl(pH 8.0), and then digested with trypsin (1∶100 enzyme/substrate) overnight at 37°C. After digestion, the enzymatic reaction was terminated using 5% acetic acid.

The enrichment of phosphopeptides was performed using a gallium-based IMAC column (Pierce, Rockford, IL), according to manufacturer's protocols with minor modification. Briefly, about 100 µg of protein digest in 5% of acetic acid was loaded onto each IMAC column. After two washes with 0.1% acetic acid and two washes with 0.1% acetic acid plus 10% acetonitrile, the bound peptides were eluted four times with 20 µl of 100 mM ammonium bicarbonate, pH 9. The resulting eluate was acidified with 5% formic acid before mass spectrometry analysis.

### Analysis of phosphopeptides by Multi-Dimensional Protein Identification Technology (MudPIT) and Linear Ion Trap-Orbitrap

The eluted peptides from each IMAC column were analyzed by one MudPIT experiment for a total of six MudPIT experiments. The MudPIT experiment was based on a previous method [29] with modifications tailored to phosphopeptide analysis. Peptides were pressure-loaded onto a 250-µm i.d. fused silica capillary column packed with a 2.5 cm long, 5 µm Partisphere strong cation exchanger (SCX, Whatman, Clifton, NJ) and a 2.5 cm, 10 µm Jupiter resin (Phenomenex, Ventura, CA), with the SCX end fritted with immobilized Kasil 1624 (PQ Corperation, Valley forge, PA). After desalting, a 100-µm i.d. capillary with a 5-µm pulled tip packed with 15 cm 4-µm Jupiter C18 material was attached to the SCX end with a ZDV union, and the entire column was placed inline with an Eksigent pump (Eksigent Technologies, Dublin, CA). Three buffer solutions used were: 5% acetonitrile/0.1% formic acid (buffer A); 80% acetonitrile/0.1% formic acid (buffer B), and 500 mM ammonium acetate/5% acetonitrile/0.1% formic acid (buffer C). Each analysis consisted of four chromatography steps. The first step consisted of a 100 min gradient from 0–100% buffer B. Steps 2–4 had the following profile: 3 min of 100% buffer A, 5 min of X% buffer C, a 10 min gradient from 0–15% buffer B, and a 130 min gradient from 15–45% buffer B, followed by a 20 min gradient increase to 100% buffer B, and a reverse of gradient to 100% buffer A. The 5 min buffer C percentages (X) were 30, 70% and 100% respectively. As peptides were eluted from the microcapillary column they were electrosprayed directly into a hybrid LTQ linear ion trap and Orbitrap (ThermoFisher, San Jose, CA) with the application of a distal 2.4 kV spray voltage. A cycle of one full-scan with 60,000 resolution at 400 *m/z* by Orbitrap (400-1400 *m/z*) followed by five data-dependent MS^2^ scan plus neutral loss-dependent MS^3^ scan by LTQ was repeated continuously throughout each step of the multidimensional separation. A precursor ion neutral loss of 98, 49 or 32 Daltons in the MS^2^ spectra was selected for further fragmentation. Normalized collision energy of 35% was used while acquiring the MS^2^ and MS^3^ spectra. The following dynamic exclusion parameters were used: repeat count -1, repeat duration – 30, list size – 100, exclusion duration – 80.

### Identification, quantification of phosphopeptides and phosphoproteins; bioinformatic analysis

MS^2^ and MS^3^ spectra were analyzed using the following software analysis protocol. Both spectra were searched with the ProLucid algorithm[30] against the rat IPI database (ftp://ftp.ebi.ac.uk/pub/databases/IPI/, version 3.17, releasing date May 18, 2006), that was concatenated to a decoy database in which the sequence for each entry in the original database was reversed. The search parameters include a static cysteine modification of 57.02146 amu and differential modification on serine, threonine and tyrosine residues of 79.9663 amu. Trypsin specificity was required for all peptides. The database search results were assembled and filtered using the DTASelect program with a spectra level false discovery rate of less than 0.5%, mass accuracy of 5 ppm. Under such filtering conditions, the estimated false discovery rate was below 1% at the peptide level.

The assembled search result file was used to obtain quantitative ratios between ^14^N (sample) and ^15^N (reference) using the software Census [31]. Census allows users to filter peptide ratio measurements based on a correlation threshold because the correlation coefficient (values between zero and one) represents the quality of the correlation between the unlabeled and labeled chromatograms and can be used to filter out poor quality measurements. In this study, only peptide ratios with correlation values greater than 0.5 were used for further analysis. For singleton analysis, we required the ^14^N/^15^N ratio to be greater than 5.0 and the threshold score to be greater than 0.5. The threshold score ranges from zero to one and represents the quality of the singleton analysis with one being the most stringent.

For Gene Ontology (GO) analysis, annotations were obtained from www.geneontology.org. Almost all nuclear proteins were annotated with multiple molecular functions. For the construction of the pie graph, the first molecular function was chosen.

For Motif analysis, we used Motif-X v1.2 (http://motif-x.med.harvard.edu/motif-x.html) [9]. We used the default settings, which include a total number of 13 characters in the motif, at least 20 occurrences of the motif in the sample input, and a p-value of 0.000001 for the selection of significant residue/position pairs in the motif. The rat IPI database was used for background analysis.

Ingenuity software was employed for global analysis [32]. The input was phosphoproteins that were 1.5 fold higher in either tissue as analyzed with Census plus phosphoproteins that were identified by at least 3 peptides in one tissue and not identified in the other tissue. The following parameters where used for the analysis. The reference set was genes from the Ingenuity Knowledge Base including all species, tissues, and cell lines. Analysis consisted of direct and indirect relationships including protein-protein interactions, microRNA-mRNA interactions, or Ingenuity Expert findings. Right-tailed Fisher's exact test was used to calculate a p-value determining the probability that each biological function and/or disease assigned to that data set is due to chance alone.

## Results and Discussion

### High confidence identification of phosphopeptides from tissue nuclear extraction

**Figure 1:**
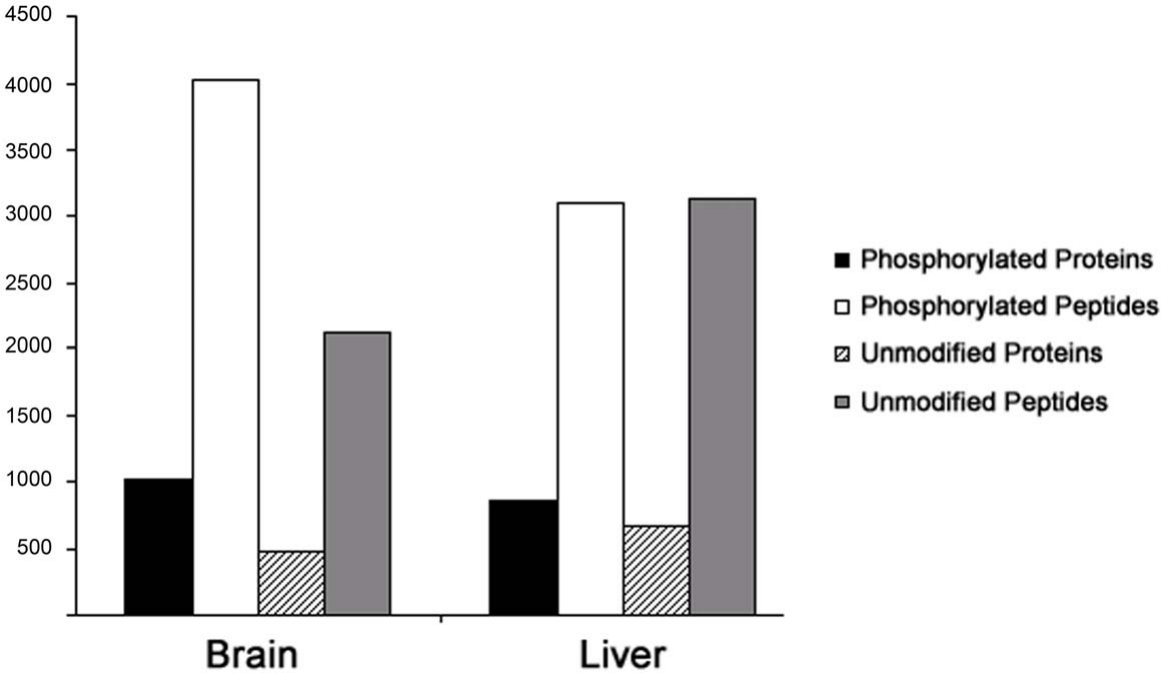
Peptide identifications from liver and brain tissue. The number (y-axis) of phosphorylated and unmodified proteins identified from brain and liver on a LTQ-Orbitrap mass spectrometer following the phosphopeptide enrichment using IMAC.

Homogenates from either liver or brain (designated ^14^N liver and ^14^N brain) of rats were mixed at a 1∶1(wt/wt) ratio with a liver homogenate from a rat labeled with ^15^N enriched diet (designated ^15^N liver). After a nuclear extraction, the samples were digested with trypsin, and the resulting peptides were applied to immobilized metal ion affinity chromatography (IMAC) column to enrich for phosphopeptides. The phosphopeptide enriched fraction was analyzed by multi-dimensional protein identification technology (MudPIT) with neutral loss dependent MS^3^ using a LTQ-Orbitrap hybrid mass spectrometer. The resulting spectra were searched with a decoy database with a final peptide false discovery rate less than 1%. We identified a total of 4028 (3433 unique) phosphorylated peptides comprising 1014 proteins from the brain analysis, and 3108 (2188 unique) phosphorylated peptides comprising 849 proteins in the liver analysis (Figure 1). For this dataset, 439 phosphoproteins and 654 unique phosphopeptides were identified in both the ^14^N brain and ^14^N liver. For unmodified proteins, we identified 471 proteins from 2123 (1680 unique) peptides in the brain, and 670 proteins from 3130 (2066 unique) peptides in the liver (Figure 1). For this dataset, 192 unmodified proteins were identified in both the ^14^N brain and ^14^N liver. Thus, IMAC was able to enrich phosphopeptides from complex tissues, and ample similarities between the protein identifications were observed to proceed with the quantification of the differences between these tissues.

Due to altered fragmentation patterns, phosphopeptides can result in less confident identifications compared to unmodified peptides. We employed a data dependent MS^3^ strategy to increase the confidence of our phosphopeptides identifications. In this strategy, a precursor ion neutral loss in the MS^2^ spectra is selected for further fragmentation, and the fragmentation pattern appears in the MS^3^ spectra. The neutral loss ions are formed by loss of phosphoric acid and are often very prominent in MS^2^ spectra. Thus, the data dependent MS^3^ is applied in phosphopeptide analysis to increase the confidence of identifications [8]. We identified 1361 phosphorylated peptides from MS^3^ spectra in the brain analysis, which confirmed 683 phosphorylated peptides from the MS^2^ identifications (Table 1). We identified 1246 phosphorylated peptides from MS^3^ spectra in the liver analysis, which confirmed 557 phosphorylated peptides from the MS^2^ identifications (Table 1). Since only a neutral loss from a phosphorylated peptide can trigger a MS^3^ event, we considered these identifications to be highly confident, and we increased the number of phosphopeptide identifications employing this MS^3^ strategy. In theory, every phosphopeptide identified in a MS^2^ spectrum should generate a higher quality MS^3^ spectrum, but in application, this is not the case for many reasons. It is most likely that the number of fragment ions in the MS^3^ scan is not large enough to identify a phosphopeptide due to the insufficient trapping of the neutral loss peptide ions. Alternatively, a MS^3^ event may not be triggered when a phosphopeptide analyzed by an MS^2^ scan does not undergo complete neutral loss of phosphate [22], or proline-directed fragmentation in MS^2^ generates ions that are more abundant than the neutral loss peptide ions. In our analysis, MS^3^ events confirmed less than 20% of our MS^2^ spectra, and similar numbers have been reported by other laboratories [8], [33].

**Table 1:** Validation of phosphopeptides identified from MS^2^ spectra with MS^3^ spectra and Debunker.

	MS^2^ peptides	MS^3^ peptides	MS^2^ = MS^3^	Debunker validated peptides
**Brain**	4028	1361	683	2934
**Liver**	3108	1246	557	2695

We also applied an in-house machine-learning computer program, Debunker [34], to validate phosphopeptide identifications derived from MS^2^ spectra. The advantage of the Debunker algorithm over the MS^3^ strategy is that it is capable of analyzing all MS^2^ spectra for features distinctive of phosphopeptides. Prominent spectral features, such as neutral loss of precursor ions, neutral loss of fragment ions, and intensity of b or y ion series, are incorporated to calculate a probability score using a support vector machine binary classification to predict the validity of the phosphopeptide identification. The predictive value from 0 to 1 is assigned to the possible phosphorylation event. A value less than 0.5 means the phosphorylation prediction is negative, while a value greater than 0.5 means the prediction is positive for a phosphorylation event. A value closer to 1 indicates the phosphorylation event is more likely to be true. Requiring a predictive value greater than 0.95, 73% of the phosphopeptides from the brain analysis and 86% of the phosphopeptides from the liver analysis were determined as a highly confident phosphopeptide (Table 1). Thus, Debunker is superior for phosphopeptide validation than the MS^3^ strategy, but the MS^3^ spectra did result in additional phosphopeptides that were not identified from the MS^2^ spectra. Finally, neither method is capable of validating phosphotyrosine peptides, which accounted for less than 5% of our phosphopeptides identifications (data not shown).

### Kinase Motif Analysis

**Figure 2:**
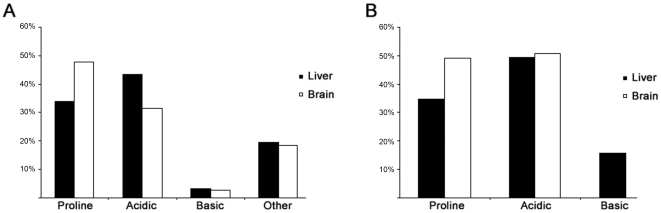
Tissue specific kinase motifs. **A**, The amino acid following the phosphorylated amino acid was categorized as proline, acidic, basic, or other. The majority of these amino acids were either proline or acidic. The y-axis represents the percentage of phosphopeptides, where the phosphorylation site could be confidently localized. **B**, The Motif-X algorithm was employed to determine if any kinase motifs existed in the data. The percentage of peptides that contained a proline, acidic, or basic residue in their motif was plotted. For peptides which contained two of these residues, they were counted in both categories. The majority of motifs contained either a proline or acidic residue.

We examined the phosphorylation site localization of the peptides that were validated by Debunker. To determine the exact amino acid that is phosphorylated can be difficult with mass spectrometry data unless only one possible phosphorylation site exists in the peptide [35]. To determine the site localization of peptides containing multiple possible phosphorylation sites, we employed a binomial probability approach that has previously been reported [35], [36]. We confidently localized the phosphorylation site in 578 and 431 unique phosphopeptides in the ^14^N brain and ^14^N liver, respectively. The most obvious characteristic of these phosphopeptides is that the majority (>75%) of these phosphorylated amino acids were followed by either proline, or an acidic residue (glutamate, or aspartate) (Figure 2A). The percentage of phosphorylation sites followed by a proline was greater in the brain, and the percentage of phosphorylation sites followed by an acidic residue was greater in the liver. To further examine these phosphopeptides, we employed the algorithm, Motif-X, to identify kinase motifs within our data[9]. When requiring a significant motif to be present at least twenty times in either brain or liver, we identified 11 and 10 motifs in the brain and liver, respectively (Table 2). Only two motifs were identified in both tissues (Figure 2B). Motif-X also computes a fold increase of the kinase motif in the sample by determining the total number of motifs found in the entire rat database. For example, the motif, PxxxKSPxxKx, occurred 27 times in the brain sample, while only 49 occurrences were observed in the entire rat database producing a fold increase greater than 1200. Consistent with this calculation, this motif was found in two annotated proteins, neurofilament M (NF-M) and neurofilament H(NF-H), which are highly abundant in brain tissue [37]. Furthermore, 12 out of the 19 observed consensus sequences have been linked to known kinases. Using different enrichment methods, a similar motif distribution was demonstrated with the nuclear extract of HeLa cells and mouse brain [8], [38], but another study has demonstrated that the yeast phosphoproteome contains more motifs with basic and other residues [15]. The observation of abundant proline motifs in the brain suggests that proline-directed kinases are more active or abundant in this tissue compared to the liver. Since it has been demonstrated that drug treatment can cause changes in percentages of proline-directed and acidic phosphopeptide motifs identified by mass spectrometry [39], the differences between liver and brain may represent differential activation of signaling systems. Consistent with the analysis of nuclear extract, the majority of the motifs observed are recognized by casein II kinase (CKII), which is mostly localized to the nucleus [40], and many CKII motifs were also observed in the phosphorylation analysis of HeLa nuclear extract indicating this nuclear kinase is very active in liver, brain, and cervix(HeLa) [8]. This corresponds to a report stating CKII has over 300 known substrates (nuclear and cytoplasmic), and it has been proposed that this kinase accounts for a significant portion of a cell's phosphoproteome [41]. Although CKII motifs were abundant in both tissues, different CKII motifs were observed in the brain and liver. This indicates that CKII may be differentially regulated, which has been previously proposed [42].

**Table 2:** Differential phosphorylation motifs identified in the liver and brain.

Motif	Peptides	Fold Increase	Tissue	Kinase	Hela
xxxxxxSxxxxxE	20	4.2	Brain	Unknown	No
xxxxxxSxDxxxx	27	5.1	Brain	CAMKII	Yes
xxxxxxSxExxxx	35	4.6	Brain	CKII	Yes
xxxxxxSDxExEx	20	128.5	Brain	CKII	Yes
xxxxxxSDExxxx	21	32.8	Brain	Unknown	No
xxxxxxSExExxx	33	21.6	Brain	CKII	Yes
xxxxxxSPxxExx	30	17.2	Brain	Unknown	No
xPxxxKSPxxxKx	27	11255	Brain	Unknown	No
xxxxxxTPxxxxx	21	6.8	Brain	Unknown	Yes
xxxxxxSxxExxx	49(B), 40(L)	4.2	Brain, Liver	CKII	Yes
xxxxxxSPxxxxx	149(B), 98(L)	5.3	Brain, Liver	ERK1, ERK2, GSK-3	Yes
xxxxxxSxxDxxx	21	4.8	Liver	CKII	Yes
xxxxxxSxxEExx	20	16.1	Liver	Unknown	No
xxxxxxSDxExxx	29	24.1	Liver	CKII	Yes
xxxxxxSDEExxx	24	117.7	Liver	CKII	Yes
xxxxxxSEEExxx	21	58.9	Liver	CKII	Yes
xxxxxDSDxxxxx	21	41.8	Liver	CKII-like	Yes
xxxRxxSxxxxxx	31	4.5	Liver	CAMKII, PKA, PKC	Yes
xxxRxxSPxxxxx	25	15.2	Liver	Unknown	Yes

The motifs were linked to the following kinases: Ca 2+/Calmodulin-Dependent Protein Kinase II (CAMKII) [35], Casein kinase II (CKII) [35], Cyclin-Dependent Kinase 5 (CDK5) [70], Extracellular Regulated Kinase 1 (ERK1) [70], Extracellular Regulated Kinase 2 (ERK2) [70], Glycogen synthase kinase 3 (GSK-3) [70], Protein Kinase A (PKA) [70], and Protein Kinase C (PKC)[ 70]. The last column denotes motifs that were also observed in Hela nuclear extracts using Motif-X [35].

### Quantification of liver and brain proteomes

**Figure 3:**
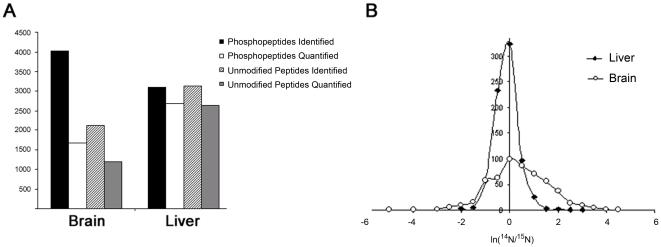
Quantification of the liver and brain proteomes. **A**, The number of phosphorylated and unmodified proteins identified and quantified from brain and liver tissue. **B**, The distribution of the N^14^/N^15^ ratios for the phosphopeptides in brain and liver tissue.

The peptides were quantified with Census, which extracts the ^14^N and ^15^N chromatograms for each peptide and determines the ^14^N/^15^N ratio using linear regression analysis [31] (Table S1). The high confidence in our phosphopeptide identifications also extended to our quantified phosphopeptides (Table 3). Greater than 80% of quantified MS^2^ peptides were validated by Debunker. The quantification efficiency (the percentage of identified peptides assigned a confident ^14^N/^15^N ratio) was dramatically different between the samples. In the liver, we observed 86.3% quantitation efficiency, and in the brain, we observed 41.7% quantitation efficiency for the phosphopeptides (Figure 3A). Since ^15^N liver was used as the internal standard, many phosphopeptides that were identified in the brain may not have a corresponding ^15^N phosphopeptide in the liver. The quantification efficiency for unmodified peptides was 56.3% and 84.0% for the brain and liver, respectively, suggesting it is indeed the choice of internal standard and not restricted to the phosphopeptide analysis (Figure 3A). We also observed a different distribution of ^14^N/^15^N ratios for proteins in the liver and brain analyses. The width of the protein ^14^N/^15^N distribution in the liver analysis was much smaller compared to the brain analysis (Figure 3B). Thus, our choice of the internal standard resulted in much larger differences quantified between brain and ^15^N liver compared to liver and ^15^N liver as expected.

**Table 3:** Validation of quantified phosphopeptides with MS^3^ spectra and Debunker.

	MS^2^ quantified	MS^2^ quantified = MS^3^	MS^3^ quantified	MS^2^ quantified validated by Debunker
**Brain**	1680	404	517	1416
**Liver**	2682	406	433	2326

### Singleton Analysis

**Figure 4:**
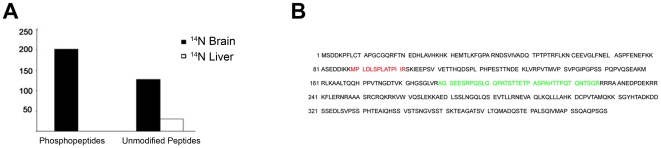
Singleton analysis. **A,** Phosphorylated and unmodified peptides determined to be singleton peptides. **B,** Three singleton phosphopeptides (green) were observed for ATF-2 with the sequence: AQS@EESRPQSLQQPATSTTETPASPAHTT@PQTQNTSGR. An identical phosphopeptide, was also assigned a N^14^/N^15^ ratio of 42.5. A different phosphopeptide (red) of ATF-2, MPLDLS@PLATPIIR was quantified with a N^14^/N^15^ ratio of 3.7 with Census.

The low quantification efficiency of the proteins from the brain analysis suggests that a ^15^N liver peptide for the corresponding ^14^N brain peptide was absent or below the limit of detection of the mass spectrometer. To retrieve this data, we performed singleton analysis on the peptides that did not pass the final filtering of Census. Census quantifies all peptides and generates a quality score, ranging from 0 to 1, to reflect the linear regression analysis of the ^14^N and ^15^N peptides. For our analysis, we required a peptide have a score greater than 0.5 for a confident correlation between the ^14^N and ^15^N peptides and to consider a peptide quantified. Scores below 0.5 may be due to noisy uninterruptible data or the detection of only one peptide and not the other (e.g. a heavy peptide is observed, but not the light or vice versa), which is described as a singleton peptide. To separate singleton peptides from noise, we required at least a 5 fold difference between the ^14^N and ^15^N peptides, and a composite score of 0.95. The composite score ranges from 0 to 1 with 1 representing a highly confident singleton peptide. In addition, there is a possibility that singleton peptides are misidentified peptides and thus, there is no corresponding peptide to be found. To avoid this possibility, we required a protein to possess at least three singleton peptides. For phosphopeptides, we observed 202 unique peptides (24 proteins) in the brain that were classified as singleton peptides, and no singleton peptides were observed in the liver (Figure 4A and Table S2). For unmodified peptides, we observed 128 unique peptides (15 proteins) in the brain that were classified as singleton peptides and 30 unique peptides (3 proteins) in the liver (Figure 4A). Although it was unexpected to find singleton peptides in the liver analysis, it may result from individual differences between animals. To verify the unmodified singleton analysis was generating accurate results, we compared our unmodified singleton proteins identified in ^14^N brain to the immunohistochemistry analysis of human tissues in the Human Protein Atlas (HPR) (http://www.proteinatlas.org). Ten out of these fifteen singleton proteins were documented in the HPR, and all ten proteins were observed to have a greater immunoreactivity in the brain compared to the liver (Table S2).

Seven proteins were documented as singleton proteins in both phosphorylated and unmodified protein analysis suggesting that the protein expression is dramatically different between liver and brain regardless of the modification. For example, calmodulin Kinase II alpha (CAMKII-alpha), has been reported to be highly expressed in the brain [43] compared to other tissues. Twenty of these singleton phosphoproteins were also quantified by Census with very large average ^14^N/^15^N ratios indicating these phosphoproteins may be *at* the limit of detection (Table S2). For example, three singleton phosphopeptides were observed for cyclic AMP-dependent transcription factor (ATF-2), and an identical phosphopeptide was assigned a ^14^N/^15^N ratio of 42.5 (Figure 4B). Interestingly, a different phosphopeptide from ATF-2 was a assigned a ^14^N/^15^N ratio of 3.7 (Figure 4B) indicating some phosphorylated sites on this transcription factor are more similar between liver and brain, while others are quite different. ATF-2 is a basic region-leucine zipper (bZIP) transcription factor and can activate transcription through cAMP response elements as a homodimer or heterodimer with members of the Jun/Fos family of transcription factors [44], [45], [46]. The ability to dimerize with a variety of proteins may result in subtle changes in DNA binding specificity [47], [48]. ATF-2 mRNA has been report to be abundant in brain compared to other adult tissues, but in liver, ATF-2 mRNA has been demonstrated to increase after a partial hepatectomy [49]. This has led to the hypothesis that ATF-2 regulates hepatocyte proliferation in the liver, but in the brain, plays a wider role in the signal transduction of differentiated neurons. The mechanism by which ATF-2 can support different functions in specific cell types is unknown. One possibility is that differential phosphorylation events can modulate the role it plays in a cell by altering its affinity for DNA or binding partners, such as c-Jun. Supporting this differential phosphorylation theory, it has been demonstrated that certain phosphorylation events within ATF-2 occur upon serum starvation while others are unaffected [50], [51]. To further complicate the regulation of ATF-2, it has been shown to be phosphorylated by multiple kinases [52], [53], [54], [55]. The novel phosphorylation site we observed to be 40-fold greater in brain is adjacent to its bZIP domain. Since this domain regulates its DNA binding specificity, it is possible that this phosphorylation event could alter the specific genes that are transcribed upon different extracellular signals, which is consistent with other transcription factors [56].

### Nuclear Proteome

**Figure 5:**
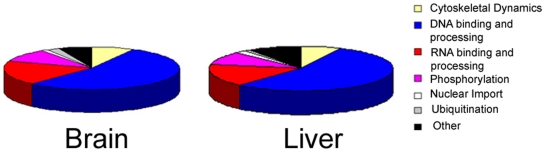
The nuclear proteome. The molecular functions annotated by GO of the quantified nuclear phosphoproteins from liver and brain.

Out of the GO annotated quantified proteins, 45% and 48% were annotated with a nuclear localization from brain and liver, respectively, with a similar distribution of molecular nuclear functions (Figure 5). This level of nuclear protein enrichment is consistent with a previous report on the nuclear proteome of brain tissue [57]. In total, there were 222 GO annotated phosphoproteins quantified in both liver and brain (Table S3). Out of these nuclear proteins, twenty-one proteins were at least 1.5 fold enriched in the brain nuclear proteome, while eighteen proteins were at least 1.5 fold enriched in the liver nuclear proteome. The nuclear phosphoprotein that was one of the most up regulated in the brain was ZFHX1B, (Zinc finger homeobox 1B, also named SIP1 and ZEB2). ZFHX1B was observed to be seven fold higher in the brain. ZFHX1B is a DNA-binding transcriptional repressor and activator [58], [59]. Although this gene is expressed in all tissues, ZFHX1B deletions cause Mowat–Wilson syndrome (MWS), which is characterized by severe mental retardation and other defects, including cardiac and urogential defects, but normal liver function [60]. The molecular mechanisms are poorly understood, but ZFHX1B has been demonstrated to be directly involved in two phosphorylation signaling pathways: Transforming growth factor beta receptor pathway [58] and the Wnt/JNK pathway [61]. Thus, we quantified two novel phosphorylation sites in the brain and liver, which may provide insight into the specific phenotype of MWS. The nuclear phosphoprotein that was one of the most up regulated in the liver was core histone macroH2A1, which was observed to be more than fourfold increase in the liver. Core histone proteins are a highly evolutionary conserved basic structural unit of chromatin with roles in DNA packaging and gene expression, however, it has been suggested that different cell types possess unique combinations of these histone proteins [62], [63]. Consistent with this theory, it has been previously reported that macroH2A1 is up regulated in rat liver compared to rat brain [64].

### Global analysis of phosphoproteomes

To identify global differences between the phosphoproteomes, we performed pathway analysis on the phosphoproteins up-regulated in these proteomes using Ingenuity Pathways Analysis (Ingenuity® Systems, www.ingenuity.com). For this analysis, we included quantified phosphoproteins with greater than 1.5 fold increase in expression compared to the other tissue and phosphoproteins that were identified by at least 3 peptides in one tissue, but not identified in the other tissue (Table S5 and Table S6). For the brain phosphoproteome, the largest cellular function represented was cellular assembly and organization with 102 of the 211 proteins analyzed designated with this function (Table S7). Many of these proteins are regulators of the cytoskeleton, which have been demonstrated to interact with the nucleus. For example, the APC (adenomatous polyposis coli) protein directly binds to the nuclear pore complex and the cytoskeleton [65]. NUMA (nuclear mitotic apparatus protein) was also identified in the category, which is a component of the nuclear matrix [66]. The nuclear matrix is a network of structural proteins analogous to the cytoplasmic cytoskeleton and hypothesized to maintain the nuclear structure and the functional subcompartments: nucleoli, speckles, and PML bodies [67]. Our data suggests that the nuclear matrix is more abundant in the brain compared to the liver. Consistent with our data, it has been reported neurons possess a more stable and larger nuclear matrix than liver hepatocytes [68]. The most significant pathway represented in our brain phosphoproteome was the PKA (protein kinase A) signaling pathway with a p-value <1.05×10^−9^ (Table S8), which measures how likely the observed association between a specific pathway and our dataset would be if it was only due to random chance. The nuclear targets of the PKA pathway up-regulated in the brain phosphoproteome were beta-catenin, histone 1 cluster protein, and ATF-2. This pathway regulates many processes in the brain, including memory and addiction [69]. For the liver phosphoproteome, the largest cellular function represented was gene expression with 61 out of the 119 phosphoproteins analyzed consisting of proteins that regulate transcription (Table S9). The most significant pathway (p-value <2.16×10^-5^) represented was farnesoid X receptor (FXR) and retinoid X receptor (RXR) activation (Table S10). FXR is a nuclear receptor that is activated by bile, which is generated in the liver. Along with RXR, FXR plays a key role in bile regulation. Overall, this global analysis reveals that the nuclear phosphoproteomes of liver and brain tissue are functionally distinct to support the different functions of these tissues.

### Conclusions

It has been proposed that differential phosphorylation between tissues may alter the function of proteins. This hypothesis may explain why many neurological diseases, such as Alzheimer's disease and Huntington's disease, are caused by mutations in ubiquitously expressed proteins, but the phenotypes are restricted to the central nervous system. Support for this hypothesis comes from a recent report demonstrating MECP2, which is mutated in the neurological disorder Rett syndrome, is phosphorylated at S421 in the brain and no other tissues tested [5]. Thus, quantitative analysis of phosphoproteomes between tissues of animal models of disease can extract novel and potential therapeutic information. Our findings provide a valuable resource as a starting point for further understanding of tissue specific gene regulation. Overall, using SILAM, we demonstrated for the first time the quantitative analysis of phosphoproteomes of different mammalian tissues.

## Supporting Information

Table S1 List of all the quantified proteins with their average ^14^N/^15^N ratios.
(XLSX)

Table S2 Singleton Analysis.
(XLSX)

Table S3 Quantified phosphoproteins common between liver and brain tissue with their GO annotations.
(XLSX)

Table S4 Quantified unmodified proteins common between liver and brain tissue with their GO annotations.
(XLSX)

Table S5 Phosphoproteins identified by at least 3 peptides in brain tissue, but not identified at all in the liver tissue.
(XLSX)

Table S6 Phosphoproteins identified by at least 3 peptides in liver tissue, but not identified at all in the brain tissue.
(XLSX)

Table S7 The top ten functions represented by the phosphoproteins from brain tissue generate by Ingenuity. The broad functional category is listed in first column followed the number of non-redundant genes designated to this category. The third column represents cellular functions which are designated to the category. The next two columns are the number of genes for each of the functions and associated p-value which measures how likely the observed association between a specific function and our dataset would be if it was only due to random chance.
(XLSX)

Table S8 The top ten functions represented by the phosphoproteins from liver tissue generate by Ingenuity.
(XLSX)

Table S9 The top ten significant pathways associated with the phosphopeptides from brain tissue with the p-value and the identified genes from the dataset which are annotated to the pathway.
(XLSX)

Table S10 The top ten significant pathways associated with the phosphopeptides from liver tissue with the p-value and the identified genes from the dataset which are annotated to the significant pathway. For this analysis, only two pathways were significant.
(XLSX)
